# Supplementary material for: Resolving the full spectrum of human genome variation using Linked-Reads
Source: Genome Res. 2019 Apr;29(4):635–45. doi: 10.1101/gr.234443.118 (PMC6442396; doi:10.1101/gr.234443.118)
Supplement: Supplemental Material [file supp_gr.234443.118_Supplemental_longranger-2.2.zip › longranger-2.2.tar.bz2!/lr2.2-public/lib/stan/cmdstan/stan/doc/api/html/math_2functions_2rising__factorial_8hpp_source.html]

Stan: src/stan/math/functions/rising\_factorial.hpp Source File


|  |  |
| --- | --- |
|  | Stan  2.5.0  probability, sampling & optimization |


- Main Page
- Related Pages
- Namespaces
- Classes
- Files

- File List
- File Members

All Classes Namespaces Files Functions Variables Typedefs Enumerations Enumerator Friends Macros Pages

- src
- stan
- math
- functions

rising\_factorial.hpp

Go to the documentation of this file.

1 #ifndef STAN\_\_MATH\_\_FUNCTIONS\_\_RISING\_FACTORIAL\_HPP

2 #define STAN\_\_MATH\_\_FUNCTIONS\_\_RISING\_FACTORIAL\_HPP

3

4 #include <stan/math/functions/log\_rising\_factorial.hpp>

5

6 namespace stan {

7  namespace math {

8

51  template<typename T1, typename T2>

52  inline typename boost::math::tools::promote\_args<T1,T2>::type

53  rising\_factorial(const T1 x, const T2 n) {

54  return std::exp(stan::math::log\_rising\_factorial(x,n));

55  }

56

57  }

58 }

59

60 #endif

log\_rising\_factorial.hpp

stan::math::log\_rising\_factorial

boost::math::tools::promote\_args< T1, T2 >::type log\_rising\_factorial(const T1 x, const T2 n)

**Definition:** log\_rising\_factorial.hpp:41

stan::math::rising\_factorial

boost::math::tools::promote\_args< T1, T2 >::type rising\_factorial(const T1 x, const T2 n)

**Definition:** rising\_factorial.hpp:53

stan::agrad::exp

fvar< T > exp(const fvar< T > &x)

**Definition:** exp.hpp:16


---

[ Stan Home Page ]

*© 2011–2014,
Stan Development Team.*
